# Development of a Scalable Extraction Process for Anthocyanins of Haskap Berry (*Lonicera caerulea*)

**DOI:** 10.3390/molecules30051071

**Published:** 2025-02-26

**Authors:** Damith Costa, H.P. Vasantha Rupasinghe

**Affiliations:** Department of Plant, Food, and Environmental Sciences, Faculty of Agriculture, Dalhousie University, Truro, NS B2N 5E3, Canada

**Keywords:** blue honeysuckle, polyphenols, cyanidin glucoside, ultrasonication, response surface method, nutraceutical

## Abstract

Haskap (*Lonicera caerulea*) berry is rich in anthocyanins, particularly cyanidin-3-*O*-glucoside (C3G). In this investigation, a response surface methodology was applied to optimize the anhydrous ethanol-based extraction parameters to obtain the maximum yield of anthocyanins from haskap berry and to compare the recovery of anthocyanins from different extraction methods. The central composite design was employed to study the effect of three independent variables (X_A_ = ultrasonic bath power, X_B_ = extraction temperature, and X_C_ = extraction time) which were found to significantly affect the response variable total anthocyanin content (TAC) and fit to the second-order polynomial model. The optimum process parameters of X_A_ = 536 W, X_B_ = 62.3 °C, and X_C_ = 63.5 min provided a predicted TAC of 16.5 mg C3G equivalence (C3GE)/g dry weight (DW), which was experimentally validated with 16.1 mg of C3GE/g DW. The optimized ultrasonication-assisted extraction process using anhydrous ethanol was also effective in recovering quercetin glycosides, catechin, procyanidin B2, and iridoids, as determined by ultra-pressure liquid chromatography–mass spectrometry. Though the anthocyanin recovery was the highest (17.6 mg of C3GE/g DW) when a deep eutectic solvent consisting of citric acid and D-(+)-maltose was used, this solvent system has limitations when preparing dehydrated extracts for industrial applications. This study concludes that the effective extraction of anthocyanins and other phytochemicals from haskap berries can be performed using food-grade anhydrous ethanol.

## 1. Introduction

Recently, consumer demand has shifted towards health-promoting, natural, and safe foods, reflecting a desire for healthier lifestyles. Therefore, the food industry is exploring innovative supplemented foods and dietary supplements with various bioactive compounds [1]. Plant-food-derived polyphenols have been gaining significant attention among health-promoting food bioactives. In addition, polyphenols have been utilized as natural food additives and food preservatives [2]. Anthocyanins, a sub-group of polyphenols, are abundant in dark-colored berries. Anthocyanin can be used as a food colorant [3] and antioxidant [4]. Therefore, food and nutraceutical industries are interested in identifying natural sources of anthocyanins. Haskap or blue honeysuckle (*Lonicera caerulea*) berry is rich in anthocyanins, particularly cyanidin-3-*O*-glucoside (C3G) [5,6]. The C3G content of haskap berry is significantly higher than that of other berries such as raspberries, blackberries, red currants, and blueberries [6,7]. C3G-rich haskap berry powder and extracts have been reported to possess various potential health benefits including antioxidant, cardioprotective, anti-inflammatory, neuroprotective, anticancer, and anti-diabetic properties [5,6,7,8,9,10].

Various extraction methods are currently being investigated to extract anthocyanins from plant sources. Anthocyanins are known to be extremely unstable in certain environmental conditions, such as in environments with light, an alkaline pH, oxygen, a high temperature, and the presence of metal ions [11]. Hence, the development of an extraction process that is appropriate for food and nutraceutical industries is crucial. The extraction process should be selected considering the cost, the high recovery of targeted bioactives, the simplicity of evaporation of the solvent, and the safety of solvent residues for human health [12]. The extraction processes under mild or non-thermal processes are required for extracting anthocyanin from haskap berries [13]. These techniques include ultrasound-assisted extraction (UAE), pressurized liquid extraction, microwave-assisted extraction, and enzyme-assisted extraction. These methods are preferred due to their ability to enhance the bioactive yield, reduce the extraction time, operate at low temperatures, minimize the degradation of bioactives during processing, and improve energy efficiency compared to conventional thermal processing [14]. UAE is a cost-effective and efficient method that is used to extract phytochemicals from plant material compared to other methods [15]. A previous study has aimed to optimize various factors, such as solvent-to-solid ratio, solvent composition, ultrasound bath temperature, and the duration of extraction, for the extraction of anthocyanin [7]. The main objective of this study is to develop a scalable, consumer-friendly process to extract anthocyanin from haskap berry to use the extracted anthocyanins in food and nutraceutical applications. Therefore, the primary focus of the present study is to establish the optimal UAE conditions that are required in order to extract anthocyanin from haskap berry using absolute ethanol by varying ultrasonic power along with extraction temperature and duration via a response surface methodology (RSM) using central composite design (CCD). The use of anhydrous ethanol as the extraction solvent is advantageous because it reduces the cost of energy for evaporation to generate dry extract compared to water-based extraction solvents. In addition, ethanol can be recovered when the extracts are dried to generate powder and can be reused for extraction.

## 2. Results and Discussion

### 2.1. Model Fitting

The central composite design was used to determine the effect of three extraction parameters (ultrasonic power, extraction temperature, and extraction time) on the total anthocyanin content (TAC) of haskap berries. The levels for each independent variable were selected based on the power range of the ultrasonic bath and the boiling point of the anhydrous ethanol (78 °C). The results of experimental runs are presented in Table 1, while the results of ANOVA are tabulated in Table 2. It is evident that the lack of fit was not significant (*p* > 0.05), indicating that the models could be applied to predict the studied responses. Also, the three-dimensional response surface plots displayed interaction effects between process parameters toward the model responses.

### 2.2. Effect of Extraction Variables on TAC

Based on the obtained responses, after the elimination of all the insignificant effects of extraction variables, the final proposed model for the TAC is shown below (Equation (1)), and the lack of fit was not significant (*p* = 0.103) with the predication of terms (R^2^ = 89.12% and Adj.R^2^ = 79.32%), indicating that the model is well fitted and reliable to explore further. Considering the linear effects, ultrasonic power (X_A_), extraction temperature (X_B_), and extraction time (X_C_) had a significant (*p* < 0.05) positive effect on TAC. Based on the quadratic model, a significant (*p* < 0.05) negative effect of extraction temperature and extraction time was observed for the yield of total anthocyanin content (TAC). The interactive effects between extraction temperature and ultrasonic power (X_B_. X_A_) had a significant (*p* < 0.05) negative effect on the TAC yield.

(1) $$\begin{array}{c} TAC\left(\frac{mgC3GE}{gDW}\right)=-7.09+0.01560\;X_{A}+0.4689\;X_{B}+0.1502\;X_{C}\\ -0.002402\;X_{B}.X_{B}-0.000645\;X_{C}.X_{C}-0.000173\;X_{B}.X_{A}\;\;\;\;\; \end{array}$$

**Figure 1:**
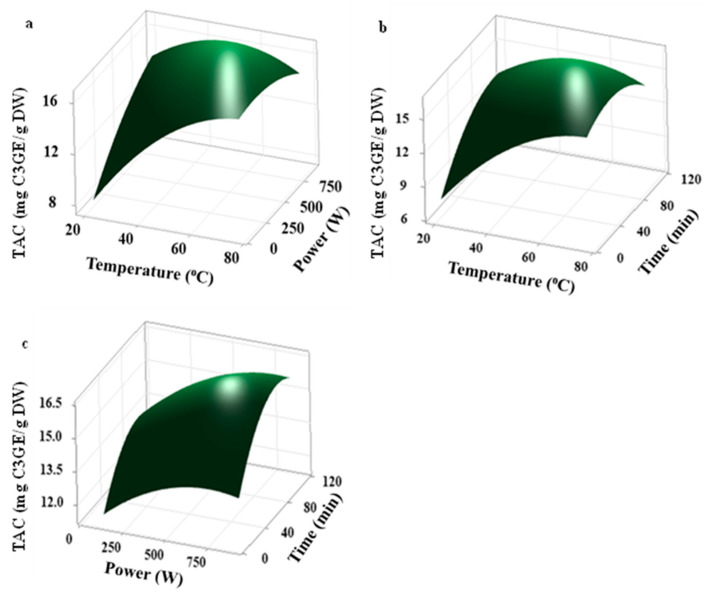
Response surface plots of the TAC (mg C3GE/g DW) extraction from haskap berry powder as a function of ultrasonic power (W), extraction temperature (°C), and extraction time (min). The following values were kept constant: (**a**) extraction time of 60 min, (**b**) ultrasonic power of 500 W, and (**c**) extraction temperature of 48 °C.

The response surface plots (Figure 1) show the interactive effects of different variables. The TAC yield for this study varied from 11.30 to 16.54 mg C3GE/g DW. The optimized extraction conditions were X_A_ = 536 W, X_B_ = 62.3 °C, and X_C_ = 63.5 min, with a predicted TAC response of 16.49 mg C3GE/g DW. For the validation of predicted conditions, an experiment was performed using the predicted optimum conditions (X_A_ = 540 W, X_B_ = 62 °C, and X_C_ = 64 min) and the actual TAC of 16.12 mg C3GE/g DW was found. The reported anthocyanin yield when using a deep eutectic solvent (DES) as the extraction solvent and an ultrasound bath temperature of 75 °C for 10 min was 19.6 mg C3GE/g DW [16]. Celli and colleagues [7] reported the total anthocyanin content of 22.73 mg C3GE/g DW when the composition consisted of 80% ethanol, 0.5% formic acid was added to the extraction solvent, and an ultrasound bath temperature of 35 °C was used for 20 min. These reported TAC values are comparatively high compared to the experimental TAC values obtained by this study.

Although this study shows high TAC extraction ultrasonic power compared to the highest reported value (510 W, from blueberry) [17], the optimum extraction temperature shows a low value compared to the value reported (75 °C) in another study using the DES as an extraction solvent [16]. Usually, when increasing the ultrasound power until a certain level, higher UAE efficiency can be obtained. However, any further increase in ultrasonication power may cause the degradation of anthocyanins. This degradation can elaborate based on the sonolysis of water stimulated by the cavitation effect during ultrasonication. The cavitation effect could produce hydroxyl radicals (^●^OH) and hydrogen peroxide (H_2_O_2_). These reactive oxygen species (ROS) cause the degradation of anthocyanin, forming chalcones by opening the ring structures [18]. When using anhydrous ethanol as an extraction solvent, the generation of ROS may be minimal compared to using other aqueous extraction solvents. Most of the anthocyanin extraction studies conducted had water in their extraction solvents, which may affect the extraction yield at elevated ultrasonication power levels. In the presence of polyphenol oxidase, anthocyanins are enzymatically degraded. This enzyme can be inactivated by heating it up to 50 °C. That way, moderately increased temperature may have a positive effect on anthocyanin retention [19].

### 2.3. Comparison of the TAC, TPC, Total Antioxidant Capacity (FRAP), DPPH^●^ Radical Scavenging Activity, and Polyphenols of Extracts Prepared from Various Extraction Processes

The low stability of anthocyanins limits their application in the food and nutraceutical industry. The acidification of the extraction solvents may increase the stability of anthocyanin via intermolecular pigmentation [20]. The low pH values, where pH < 6, are beneficial to maintaining the stability of anthocyanins, and the addition of organic acids could improve the anthocyanin stability [21]. The use of DES as an extraction solvent has recently sparked interest as it is a green extraction solvent [14]. Table 3 shows the variation in the TAC and anthocyanin profiles with five different extraction solvents under optimized conditions. In this study, the highest C3G yield was provided by the DES used as the extraction solvent (24.9 mg/g DW), 2% acetic acid or 2% citric acid with anhydrous ethanol, and anhydrous ethanol alone. However, measuring the TAC by pH differential methods shows a significantly higher amount of total anthocyanins in DES extraction compared to the optimized anhydrous ethanol, and deionized water/UAE provided the lowest TAC.

**Figure 2:**
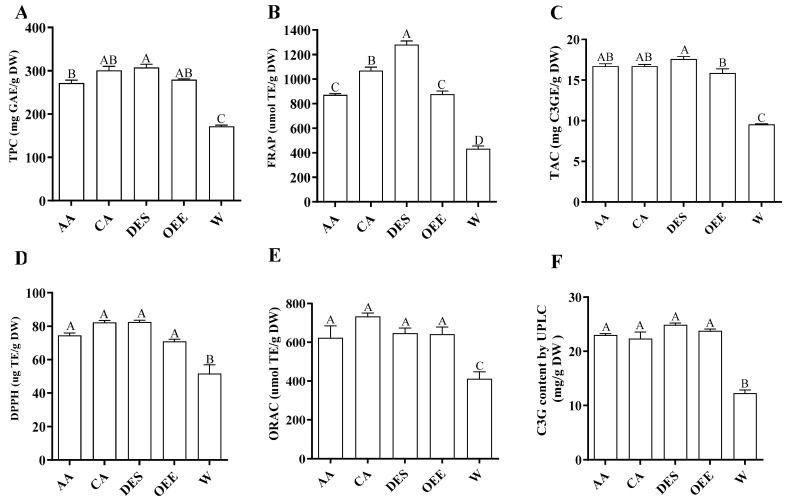
The TPC (**A**), FRAP (**B**), TAC (**C**), DPPH (**D**), ORAC (**E**), and C3G (**F**) content variations of different extraction processes. Abbreviations: C3GEs, C3G equivalents, OEE, optimum extraction condition using anhydrous ethanol and UAE; AA, anhydrous ethanol with 2% acetic acid/UAE); CA, anhydrous ethanol with 2% citric acid/UAE; DES, deep eutectic solvent system with UAE; W, deionized water with UAE. The different letters of means (A–D) indicate the significant differences (*p* > 0.05) among them.

Among the non-anthocyanin polyphenols investigated, Q3R and chlorogenic acid were detected in relatively high quantities in the five extracts (Table 4). The recovery of Q3R was greater in all anhydrous ethanol extraction processes compared to water extractions. The addition of 2% acetate or 2% citrate to absolute ethanol did not significantly increase the TPC of the extracts (Figure 2A).

The DPPH radical scavenging activity of different extraction methods was investigated based on the inhibition percentage (Figure 2D). When water was used as the extraction solvent, it showed the least antioxidant activity and when the DES was used as the extraction solvent, the highest antioxidant activity of the anthocyanin was observed. However, the DPPH radical scavenging activity of the DES was not significantly different from that of extracts prepared using 2% acetic acid in ethanol and 2% citric acid in ethanol. When water was used as the extraction solvent, it showed the least TAC values (Figure 2C). However, the ORAC value of optimized anhydrous ethanol extract was not significantly different from that of extracts prepared using 2% acetic acid in ethanol and 2% citric acid in ethanol and the DES.

C3G comprises about 80% anthocyanins of haskap beery [5]. C3G is found in almost all North American berries. C3G content is significantly greater in haskap berries compared to other common berries such as strawberries, blueberries, cranberries, and chokeberries [6]. The total phenolic content of haskap berry was reported to be the highest (1111 mg GAE/100 g FW) among all other North American berry types [22].

Anhydrous ethanol was effective in recovering polyphenols with a relatively higher antioxidant capacity than water (Figure 2). In general, aqueous ethanol has been commonly used for the extraction of polyphenols from plant materials [23]. Celli and colleagues [7] extracted C3G, C3R, and Peo3G using 80% aqueous ethanol with 0.5% formic acid as the extraction solvent. Since the objective of this study was to develop a scalable, consumer-friendly process to extract anthocyanin from haskap berries, it is mandatory to use a solvent or a solvent mixture that is not toxic to human health. Residues of toxic organic solvents are a major concern in food and nutraceutical industries. The ingestion of formic acid in the digestive tract of humans can cause ulcers and formic acid to metabolize to formaldehyde [24]. As a solvent that is generally recognized as safe (GRAS), anhydrous ethanol [25] shows relatively low toxicity toward human health when compared to organic solvents such as methanol, acetone, hexane, formic acid, and acetonitrile [26,27]. For the commercial production of dried food ingredients or nutraceuticals, the evaporation of extracts is required [28]. Due to the low boiling point of anhydrous ethanol, it can be easily evaporated with a low energy cost compared to water or aqueous ethanol-based extracts [29]. The maltose of DES-based extracts in supplemented food could be a negative factor for consumers with type 2 diabetes [30].

### 2.4. Correlation Analysis of the TAC, TPC, C3G, ORAC, DPPH, and FRAP of Haskap Berry Extracts

**Figure 3:**
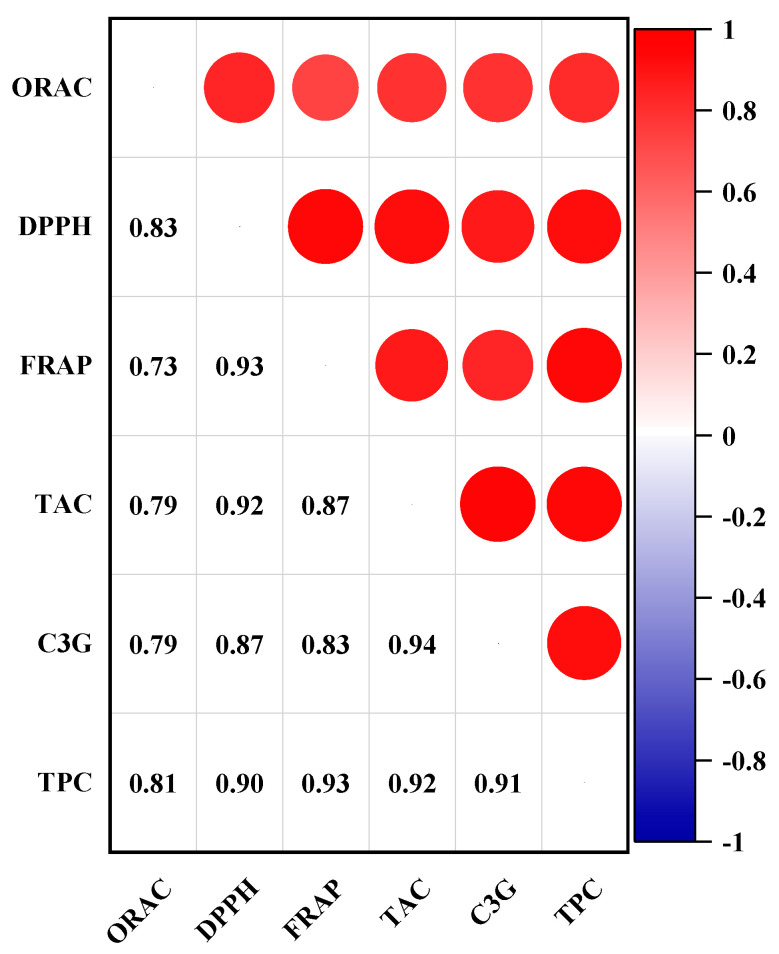
Correlation coefficients among the TAC, TPC, C3G, ORAC, DPPH, and FRAP of haskap berry extracts prepared from various methods (n = 15).

To understand the correlations among the measured parameters, the Pearson correlation analysis of the TAC, TPC, C3G, ORAC, DPPH, and FRAP was performed (Figure 3). The strongest correlation can be seen between the TAC and TPC, which agrees with a previous study [31]. A strong correlation between the TAC and C3G was also observed, demonstrating the contribution of C3G to total anthocyanins. A similar correlation has been shown by Khattab et al. [32]. Strong correlations were shown between the TPC and three antioxidant capacity assays (ORAC, FRAP, and DPPH). DPPH results showed a strong correlation with the FRAP, TAC, and TPC. Antioxidant capacity from the FRAP showed a strong correlation (R = 0.93) with the TPC, but the ORAC correlation with the FRAP was less strong (R = 0.73) compared to the correlation between other antioxidant assays. Differences in antioxidant capacities in different assays may be attributed to the variations in the mechanisms underlying the assays [33].

### 2.5. Iridoid Content of Haskap Berry Extracts

Iridoids are potent with many biological activities, such as anti-inflammatory, anti-tumor, hepatoprotective, neuroprotective, and cardioprotective properties [34]. Structurally, iridoids can be divided into four groups: iridoid glycosides, secoiridoid glycosides, non-glycosidic iridoids, and bis-iridoids. Iridoids investigated in this study mainly belong to iridoid glycosides (loganin and loganic acid), and secoiridoid glycoside (sweroside) [34].

The highest total iridoids (loganin, loganic acid, and sweroside) were achieved using deionized water and a DES as the extraction solvents (Table 5). The recovery of loganin was not influenced by the different extraction methods used in this study. In contrast, sweroside extraction was most effective using the DES, which may be due to increased solvent polarity by citric acid. Similar to our findings, loganic acid was the most abundant iridoids in haskap berries, representing 22% to 73% of the total iridoids [35]. Hot water extraction recovered more iridoids than ethanol or methanol [36]. While polyphenols are prevalent in fruits, iridoids are relatively rare, exceptions include cranberry [37], bilberry [38], and haskap berries [35]. Cornelian cherry contains a wide range of iridoid content from 87 to 493 mg/100 g FW [39]. In 13 wild blueberry genotypes, iridoids range from 2.0 to 237 mg/100 g DW [40]. Bilberry contains 13 mg/100 g FW [41]. The previously reported iridoid contents of haskap berry grown in Poland are much higher (120 to 276 mg/100 g FW) [35] compared to the Canadian-grown haskap berries used in this study.

### 2.6. Single-Factor Experiment—The Effect of Ethanol and Ultrasonication Power on Anthocyanin Extraction

**Figure 4:**
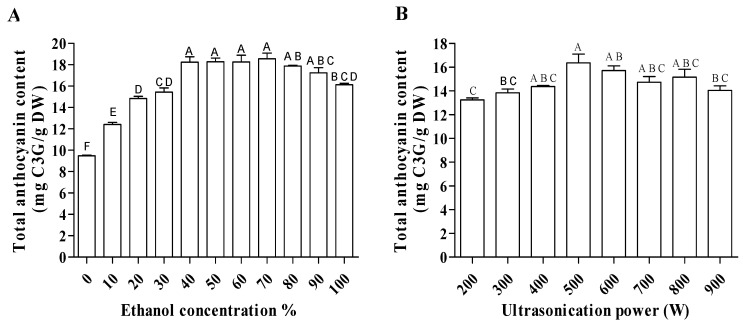
TAC of haskap berry extracts in response to different ethanol concentrations (**A**) and different ultrasonic power levels (**B**). The different letters of means (A–D) indicate the significant differences (*p* > 0.05) among them.

When water was used as an extraction solvent, the lowest amount of anthocyanins was recovered (Figure 4A). Ethanol percentage ranging from 40% to 90% recovered the highest amount of anthocyanins. A similar pattern of recovery of anthocyanins was observed by Cacace et al. [42] when extracting anthocyanin from black currant berries. The electric charge changes with the addition of ethanol are responsible for this behavior. The extraction yields of polyphenols were affected by the concentration of ethanol in the solvent. When the ethanol concentration is high, the dielectric constant is low, so it lowers the energy required to break the water arrangement [43]. Therefore, nonpolar flavanols needed a higher ethanol concentration (75% to 100%) to obtain a high recovery. For polar phenolic acids, the optimum concentration of ethanol was 70% to 90%. On the other hand, a mixture of ionic anthocyanins (flavylium cation, AH^+^) did not require a high ethanol concentration, and the maximum yield can be obtained at approximately 50% ethanol [42].

The highest TAC can be obtained using the ultrasonication power ranging from 400 W to 800 W (Figure 4B). Very few studies have investigated the impact of ultrasonication power on anthocyanin recovery. One study showed that 400 W gives the maximum anthocyanin recovery from *Rubia sylvatica* [44]. Liao et al. [45] described 200 W as the power which contributes to the highest anthocyanin yield from purple eggplant peel. This study was carried out using a power range of 100 W to 300 W. Our study shows that increasing the power above 400 W is not necessary in terms of energy cost for the process, as the anthocyanin yield is not significantly different when ultrasonic power of 400 W is used.

### 2.7. TAC Recovery Percentage at Optimum Extraction Conditions

The recovery percentage of the TAC from haskap powder was performed in subsequent different extraction attempts using the same optimum conditions until anthocyanin color was not visually observed in the supernatant (Table S1). Based on the TAC determinations, the first attempt recovered about 89.0% of the potential total TAC. Interestingly, 97.9% of the TAC can be recovered by conducting two subsequent extractions and pooling them. The above observation indicates that the optimized extraction process recovers about 90% of extractable anthocyanins from dehydrated haskap berry powder.

## 3. Material and Methods

### 3.1. Plant Materials, Chemicals, Reagents, and Equipment

The fully mature haskap berry variety ‘Tundra’ of 2022 harvest was obtained from Atlantic Haskap, Elderbank, NS, Canada. Gallic acid, sodium carbonate, sodium acetate trihydrate, 2,4,6-tris(2-pyridyl)-*S*-triazine (TPTZ), Folin–Ciocalteu reagent, ferric chloride, 6-hydroxy-2,5,7,8-tetramethylchroman-2-carboxylic acid (Trolox), D-(+)-maltose, citric acid, glacial acetic acid, and 1,1-diphenyl-2-picrylhydrazyl (DPPH) were obtained from Sigma-Aldrich (Oakville, ON, Canada). Anhydrous ethanol was purchased from Commercial Alcohols, Brampton, ON, Canada. The extraction process was conducted in an ultrasonic bath with a fixed frequency of 40 kHz (Model 4HT-1524-12, Crest, Ewing, NJ, USA). The temperature was controlled within ±1 °C with a calibrated thermometer and adjusted with cold water.

### 3.2. Experimental Design

The experiment was conducted in two stages. In the first stage, an RSM was selected for the optimization of process parameters to maximize the yield of total anthocyanin content (TAC) from freeze-dried haskap berry powder (particle size 0.1 to 10 μm), which was prepared using a freeze dryer (Kinetics, FTS Systems Inc., Stone Ridge, NY, USA) at −40 °C for 72 h and then ground using a standard coffee grinder. A central composite design was specifically chosen to study the effect of three independent variables (X_A_ = ultrasonic bath power (W), X_B_ = extraction temperature (°C), and X_C_ = extraction time (min)) on the response variables (Y) of the TAC (mg C3GE/g DW). The ranges of independent variables used for the optimization study were as follows: X_A_—100 W to 900 W, X_B_—20 °C to 76 °C, and X_C_—10 min to 110 min. This study explored the effect of these variables on anthocyanin extraction using a solvent-to-solid ratio of 25:1 mg/mL [7] and a solvent composition consisting of anhydrous ethanol. A level-three factor central composite design, consisting of 20 experimental runs, was performed, and the observed values are given in Table 1. Response surface analysis and analysis of variance (ANOVA) were used to determine the regression coefficients and the statistical significance of the model terms. The observed values were then fitted into a second-order polynomial model, as shown in Equation (2), and the regression coefficient β was generated, as shown in Table 2.

(2) $$Y=\beta_{0}+Ʃ\beta_{i}X_{i}+Ʃ\beta_{ii}X_{i}2+Ʃ\beta_{ij}X_{ij}$$

Y indicates the response variable; X_i_ and X_j_ are independent variables; β_0_ is the constant coefficient of the model; and β_i_, β_ii_, and β_ij_ are the regression coefficients of the single effects, quadratic effects, and interactive effects of independent variables, respectively.

In the second phase used for drawing comparisons with anhydrous ethanol (AE), four different extraction solvents, 2% acetic acid in anhydrous ethanol (AA), 2% citric acid in anhydrous ethanol (CA), water (W), and deep eutectic solvent (DES) consisting of citric acid and D-(+)-maltose [16], were used with the same optimal parameters of this study. The second-phase extracts were analyzed to identify the TAC, the total phenolic content (TPC), and total antioxidant capacity using the ferric-reducing antioxidant power (FRAP) assay, the DPPH radical scavenging assay, the oxygen radical absorbance capacity assay (ORAC), and polyphenol profiling using ultra-pressure liquid chromatography (UPLC) with electrospray ionization (ESI) and mass spectrometry (MS) [5].

### 3.3. Validation of the Model

The optimized conditions were validated by measuring the TAC of extracts prepared based on the optimum conditions predicted using an RSM in Minitab^®^ (v. 20). The experimental values were compared with predicted values to determine the validity of the model.

### 3.4. UAE of Anthocyanins

UAE was conducted in 50 mL amber glass vials, where 0.5 g of freeze-dried haskap berry powder was transferred, and 12.5 mL of extraction solvent was added to each vial. The vials were vortexed for 10 s and placed in the ultrasonic bath for each extraction condition. Following extraction, samples were centrifuged (Sorvall ST 16, Thermo Scientific, Darmstadt, Germany) at 3000× *g* for 5 min. The supernatant was then filtered through a 0.45 µm syringe filter and stored in 15 mL Falcon tubes in the dark at −20 °C.

### 3.5. Determination of Total Monomeric Anthocyanin Content (TAC)

The TAC of samples was determined in triplicate using the pH differential method (AOAC method 2005.02) using a UV–visible spectrophotometer (Tecan Infinite^®^ M200 PRO, Morrisville, NC, USA). Thirty-times-diluted samples in both pH 1 and pH 4.5 buffer were prepared, and the absorbance (A) was measured at 520 nm and 700 nm.

The A of the extract was calculated using the following equation:

(3) A=(A520–A700) at pH 1.0−(A520–A700) at pH

The TAC was calculated using the following equation:

(4) TAC=(A×M×DF×103)/(ε×L)

This equation shows the molar extinction coefficient (ε), 26,900; the molecular weight of C3G (MW), 484.83 g/mol; the dilution factor (DF); and the path length (L) [46]. Results are expressed in mg of C3G equivalents per g of the dry weight (mg C3GE/g DW) of haskap berries.

### 3.6. Determination of Polyphenols of the Optimized Extract Using UPLC-ESI-MS Analysis

The most prominent polyphenols present in the haskap extracts were identified and quantified [5] using UPLC-ESI-MS (Quattro Micro, Waters, Milford, MA, USA). Briefly, extracts were filtered through 0.45 μm nylon syringe filters. For the separation of analytes, an Aquity BEH C_18_ (100 mm × 2.1 mm, 1.7 μm) column (Waters, Milford, MA, USA) was used with an ESI in positive ion mode (ESI+), with a capillary voltage of 3000 V and a nebulizer gas (N_2_) temperature of 375 °C. A flow rate of 0.2 mL/min using 2% formic acid in water (A) and 2% formic acid in methanol was used. A linear gradient was used with the following proportions of solvent B (time in min [t], B%): (0, 10%), (7, 30%), (16, 40%), (17, 40%), (18, 90%), and (20, 10%). The anthocyanins (M + H^+^) were identified using single-ion monitoring (SIM) mode: *m*/*z* 448.8 for cyanidin-3-glucoside, *m*/*z* 610.8 for cyanidin-3,5-diglucoside, *m*/*z* 462.8 for peonidin-3-glucoside, and *m*/*z* 594.8 for cyanidin-3-rutinoside. The analyses of non-anthocyanins (other polyphenols and iridoids) were performed using ESI in negative ion mode (ESI−), with a capillary voltage of 3000 V and a nebulizer gas (N_2_) temperature of 375 °C. The same column was used with a flow rate of 0.3 mL/min and a gradient mobile phase using 0.1% formic acid in water (A) and 0.1% formic acid in acetonitrile (B). Solvent B was applied at time t (min) for the following (t, B%): (0, 6%), (6, 20%), (8, 80%), (9, 80%), and (10, 6%). The following analytes were identified using SIM mode: *m*/*z* 300.7 for quercetin (Q), *m*/*z* 462.5 for Q-3-glucoside, *m*/*z* 608.5 for Q-3-rutinoside, *m*/*z* 594.7 for Q-3-arabinoglucoside (peltatoside), *m*/*z* 288.7 for catechin, *m*/*z* 577.1 for procyanidin B2, *m*/*z* 352.7 for chlorogenic acid, *m*/*z* 435.1 for loganin, *m*/*z* 375.1 for loganic acid, and *m*/*z* 403.1 for sweroside. All the analytes were quantified using calibration curves generated using external standards. The limit of detection of all the analytes was between 0.01 and 0.1 mg/L.

### 3.7. Determination of Total Phenolic Content (TPC)

The TPC in extracts was measured using the Folin–Ciocalteu (FC) assay, as described by Huber et al. [47]. Gallic acid was used for the generation of a standard curve using the extraction solvent (anhydrous ethanol) and diluted from concentrations of 59 to 1470 μM. The solutions were made fresh under reduced light conditions and the reaction was carried out under dark conditions. Then, 20 mL of the diluted extract or gallic acid standard was mixed with 100 μL of the 0.2 N Folin–Ciocalteu phenol reagent in 96-well, clear, polystyrene microplates (COSTAR 9017) and gently mixed. After 5 min, 80 μL of 7.5% (*w*/*v*) sodium carbonate was added to each well and mixed. The mixture was incubated for 2 h at ambient temperature before the absorption was measured at 760 nm using the Tecan Infinite^®^ M200 PRO (Morrisville, NC, USA). Results were expressed as mg of gallic acid equivalents per g of dry weight (mg GAE/g DW).

### 3.8. The Ferric-Reducing Antioxidant Power (FRAP) Assay

The FRAP assay was performed according to Benzie et al. [48] with some modifications. The reaction reagent (FRAP solution) was made immediately before the assay by mixing 300 mM acetate buffer (pH 3.6), 10 mM TPTZ solution, and 20 mM ferric chloride solution in a ratio of 10:1:1. The Trolox standard solution was prepared by dissolving 0.025 g of Trolox in 100 mL of anhydrous ethanol to make 1 mM Trolox. A calibration curve was developed using Trolox stock solution with appropriately anhydrous ethanol dilutions (from 5 to 450 μM Trolox concentrations). Both the FRAP solution and the samples in the microplate were warmed to 37 °C prior to the assay. The FRAP analysis was performed by reacting 20 μL of blank or standard sample with a 180 μL FRAP solution in 96-well clear polystyrene plates (COSTAR 9017). Absorption was measured at 593 nm using the Tecan Infinite^®^ M200 PRO (Morrisville, NC, USA). FRAP values were expressed as μmol Trolox equivalents per g of the sample dry weight (μmol TE/g DW).

### 3.9. DPPH Radical Scavenging Assay

The free radical scavenging activity of different anthocyanin extracts was measured using the method described by Guo et al. [49] with slight modifications. In short, the DPPH solution was made by dissolving 7.9 mg of DPPH in 100 mL of methanol and stored at −20 °C. The DPPH solution (100 μL) and the sample (100 μL) were mixed in a 96-well plate and kept in a 37 °C incubator for 30 min. The absorbance was measured at 520 nm using the Tecan Infinite^®^ M200 PRO (Morrisville, NC, USA). The Trolox standard stock solution was prepared by dissolving 0.25 mg of Trolox in 100 mL of the anhydrous ethanol to make 2.5 mg/L Trolox. A calibration curve was developed using the Trolox stock solution with appropriate dilutions (from 0.781 to 25 μg/L Trolox concentrations). The following equation was used to calculate the inhibition percentage:

(5) $$Antioxidant\;activity\;\left(\%inhibition\right)=\left(\frac{Ab\;blank-Ab\;sample}{Ab\;blank}\right)\times 100$$

where Ab sample is the absorbance value of different anthocyanin extracts and Ab blank is the absorbance value of the anthocyanin-free extract solvent. The antioxidant activity was expressed as μg Trolox equivalents per g of the sample dry weight (μg TE/g DW).

### 3.10. Oxygen Radical Absorbance Capacity (ORAC) Assay

The ORAC assay was performed using a previously established protocol [50]. The samples and standards of the fluorescein sodium salt (0.957 mM) were dissolved in 75 mM phosphate buffer (K_2_HPO_4_/NaH_2_PO_4_, pH 7). From the sample or Trolox standard, 35 µL and 130 µL volumes of the fluorescein probe were combined in black 96-well polystyrene microplate wells and the plate was warmed to 37 °C for 10 min. After incubation, 35 µL of 150 mM pre-warmed (37 °C) AAPH was transferred into the wells. The plate was maintained at 37 °C for the duration of the analyses (approximately 200 min) with excitation and emission readings every 60 s. Excitation of the reaction mixture was carried out at 490 nm and the emission was read at 540 nm. The antioxidant capacity of the samples was calculated as Trolox equivalents using a quadratic relation developed from the area under the fluorescence decay curves for standards made from 10 to 150 µM concentrations. The antioxidant activity was expressed as μmol Trolox equivalents per g of the sample dry weight (µmole TE/g DW).

### 3.11. Single-Factor Experiment

The effect of ethanol percentage and ultrasonication power on the TAC of extracts was studied using single-factor experiments. One factor was changed while the other factors were fixed in each experiment at optimum extraction conditions (540 W, 62 °C, and 64 min). The effect of ultrasound power was studied at 200–900 W while the other factors (a temperature of 62 °C and a time of 64 min) remained fixed. In the same manner, the ethanol concentration from 0% to 100% was investigated at 540 W, 62 °C, and 64 min. The extracts were centrifuged, and the supernatants were tested for the TAC.

## 4. Conclusions

This study investigated the optimized conditions required for the recovery of anthocyanins from freeze-dried haskap berry powder using anhydrous ethanol as an extraction solvent. Optimized conditions for UAE for total anthocyanin extraction were a temperature of 62.3 °C, an extraction time of 63.5 min, and an ultrasonic power of 536 W. The validity of the model obtained by the RSM was demonstrated by the similar extraction yield to the predicted response (16.5 mg C3GE/g DW) with the actual experimental result (16.1 mg C3GE/g DW) under optimum extraction conditions. The addition of citric acid or acetic acid to the anhydrous ethanol does not affect the extraction yield of C3G from haskap berry powder under the optimized conditions. The emerging DES containing maltose and citric acid as an alternative extraction process is as effective as optimized anhydrous ethanol extraction; however, the DES has limitations in scale-up extractions for commercial applications. Compared to traditional solvents such as methanol, food-grade ethanol has a low environmental impact due to fast biodegradation and is less harmful to the environment. The findings of the current study are useful as they encourage the food and nutraceutical industry to use food-grade anhydrous ethanol as a cost-effective and green extraction solvent to extract and prepare C3G-rich bioactive preparations from haskap berries.

## Figures and Tables

**Table 1:** Central composite design with coded and actual values for each investigated factor and resultant total anthocyanin content (TAC) (Y in mg C3GE/g DW of haskap berry powder).

Run No.	X_A_	X_B_	X_C_	Y
1	500 (0)	48 (0)	60 (0)	15.8
2	500 (0)	48 (0)	60 (0)	16.3
3	500 (0)	48 (0)	110 (+1.68)	15.9
4	500 (0)	48 (0)	10 (−1.68)	12.1
5	260 (−1)	31 (−1)	90 (+1)	12.0
6	740 (1)	65 (1)	90 (+1)	15.2
7	500 (0)	48 (0)	60 (0)	16.5
8	740 (1)	65 (1)	30 (−1)	16.5
9	740 (1)	31 (−1)	30 (−1)	12.9
10	500 (0)	48 (0)	60 (0)	15.3
11	500 (0)	76 (+1.68)	60 (0)	15.6
12	900 (+1.68)	48 (0)	60 (0)	16.1
13	260 (−1)	65 (1)	90 (+1)	16.0
14	100 (−1.68)	48 (0)	60 (0)	13.5
15	260 (−1)	31 (−1)	30 (−1)	11.3
16	260 (−1)	65 (1)	30 (−1)	16.0
17	500 (0)	20 (−1.68)	60 (0)	11.9
18	500 (0)	48 (0)	60 (0)	15.2
19	740 (1)	31 (−1)	90 (+1)	15.5
20	500 (0)	48 (0)	60 (0)	15.9
Predicted optimum conditions	536	62.3	63.5	16.5 *

* Predicted TAC.

**Table 2:** ANOVA results of significant factors in the quadratic model.

Source	df	Adj SS	Adj MS	F-Value	*p*-Value
Model	9	53.01	5.889	9.10	0.001
Linear	3	35.88	11.96	18.5	0.000
X_A_	1	6.074	6.074	9.38	0.012
X_B_	1	24.61	24.61	38.0	0.000
X_C_	1	5.196	5.196	8.03	0.018
Quadric	3	10.56	3.521	5.44	0.018
X_B_. X_B_	1	6.369	6.369	9.84	0.011
X_C_. X_C_	1	4.708	4.708	7.27	0.022
Two-way interaction	3	6.561	2.187	3.38	0.062
X_B_. X_A_	1	3.736	3.736	5.77	0.037
Error	10	6.472	0.647		
Lack of fit	5	4.997	0.999	3.39	0.103
Pure error	5	1.476	0.295		
Total	19	59.48			

**Table 3:** Anthocyanin concentrations measured by UPLC-ESI-MS and the pH differential assay of haskap berry extracts prepared using different extraction processes.

Extraction Process	Anthocyanins (mg/g DW) by UPLC-ESI-MS				
	C3G	C35DG	Peo3G	C3R	TAC (mg C3GE/g DW)
Optimum extraction conditions using anhydrous ethanol and UAE (OEE)	23.8 a	0.94 b	1.25 a	1.43 ab	15.9 b
Anhydrous ethanol with 2% Acetic acid/UAE	23.0 a	0.96 b	1.16 b	1.39 b	16.7 ab
Anhydrous ethanol with 2% Citric acid/UAE	22.3 a	0.99 b	1.18 ab	1.43 ab	16.7 ab
DES/UAE	24.9 a	1.13 b	1.16 b	1.57 a	17.6 a
Deionized water/UAE	12.3 b	1.48 a	0.67 c	1.18 c	9.56 c

Mean values of four types of anthocyanin obtained by means of UPLC-ESI-MS analysis (C3G, C35DG, Peo3G, and C3R) (mg/g DW) and the total anthocyanin content measured using the pH differential method (mg C3GE/g DW). Within each column, means sharing the same letters (a–c) are not significantly different (*p* < 0.05). Abbreviations: C3G, cyanidin-3-*O*-glucoside; C3GE, C3G equivalents, C35DG, cyanidin-3,5-diglucoside; C3R, cyanidin-3-*O*-rutinoside; DES, deep eutectic solvent system; Peo3G, peonidin-3-*O*-glucoside.

**Table 4:** Flavonoid and chlorogenic acid content of haskap berry extracts prepared from different extraction processes.

Extraction Process	Flavonoids Concentration by UPLC-ESI-MS (mg/g DW)						
	Q3G	Q3ArG	Quercetin	Q3R	Catechins	PB2	ChlA.
Optimum extraction conditions using anhydrous ethanol/UAE (OEE)	0.33 a	0.13 b	0.01 ab	1.73 b	2.14 b	0.10 a	1.69 a
Anhydrous ethanol with 2% acetic acid/UAE	0.26 b	0.11 b	0.01 ab	1.44 c	2.37 b	0.09 a	1.76 a
Anhydrous ethanol with 2% citric acid/UAE	0.35 a	0.15 a	0.02 a	2.00 a	2.82 a	0.11 a	1.84 a
DES/UAE	0.22 b	0.13 b	0.01 bc	1.41 c	3.17 a	0.09 a	1.93 a
Deionized water/UAE	0.13 c	0.11 b	0.00 c	1.22 c	0.98 c	0.08 a	1.33 b

Mean values of different flavonoids quantified by means of UPLC-ESI-MS analysis (mg/g DW). Within each column, means sharing the same letters (a–c) are not significantly different (*p* < 0.05). Catechins are composed of epicatechin and catechin. Abbreviations: DES, deep eutectic solvent system; Q3G, quercetin-3-*O*-galactoside; Q3ArG, quercetin-3-arabinoglucoside (peltatoside); Q3R, quercetin-3-*O*-rutinoside; PB2, proanthocyanin B2; ChlA., chlorogenic acid; and TPC, total phenolic content.

**Table 5:** Iridoid content of haskap berry extracts.

Extraction Process	Iridoids Concentration by UPLC-ESI-MS (mg/g DW)			
	Loganin	Loganic Acid	Sweroside	Total Iridoids
Optimum extraction conditions using anhydrous ethanol/UAE (OEE)	0.23 a	1.27 b	0.23 d	1.72 b
Anhydrous ethanol with 2% acetic acid/UAE	0.21 a	1.22 b	0.23 cd	1.67 b
Anhydrous ethanol with 2% citric acid/UAE	0.22 a	0.60 c	0.26 bc	1.08 c
DES/UAE	0.22 a	1.93 a	0.31 a	2.46 a
Deionized water/UAE	0.20 a	1.94 a	0.26 bc	2.41 a

Mean values of most abundant iridoids (loganin, loganic acid, and sweroside) measured by UPLC-ESI-MS analysis (mg/g DW). Within each column, the means sharing the same letters (a–d) are not significantly different (*p* < 0.05). Total iridoids are composed of loganin, loganic acid, and sweroside. Abbreviations: DES, deep eutectic solvent system.

## Data Availability

Data are contained within the article.

## Supplementary Materials

The following supporting information can be downloaded at the following: https://www.mdpi.com/article/10.3390/molecules30051071/s1, Tabel S1. Anthocyanin recovery percentage at optimum extraction condition.
