# Supplementary material for: Development of a Scalable Extraction Process for Anthocyanins of Haskap Berry (Lonicera caerulea)
Source: Molecules. 2025 Feb 26;30(5):1071. doi: 10.3390/molecules30051071 (PMC11902241; doi:10.3390/molecules30051071)
Supplement: Supplementary file 1 [file molecules-30-01071-s001.zip › molecules-3463352-supplementary.pdf]

Development of a Scalable Extraction Process for Anthocyanins of Haskap Berry (*Lonicera caerulea*)

Damith Costa and H.P. Vasantha Rupasinghe*

Supplementary Data

Tabel S1. Anthocyanin recovery percentage at optimum extraction condition

Extraction cycle	TAC (mg C3GE/g DW)	Yield % (TAC)
1	15.43 ± 0.19	89.0
2	1.55 ± 0.14	8.91
3	0.25 ± 0.02	1.45
4	0.11 ± 0.01	0.63
Total	17.34	

TAC. Total anthocyanin content (mean ± standard deviation); C3GE, cyanidin-3-glucoside equivalents
